# Taking an educational psychology course improves neuroscience literacy but does not reduce belief in neuromyths

**DOI:** 10.1371/journal.pone.0192163

**Published:** 2018-02-05

**Authors:** Soo-hyun Im, Joo-Yun Cho, Janet M. Dubinsky, Sashank Varma

**Affiliations:** 1 Department of Educational Psychology, University of Minnesota, Minneapolis, MN, United States of America; 2 Department of Elementary Education, Seoul National University of Education, Seoul, South Korea; 3 Department of Neuroscience, University of Minnesota, Minneapolis, MN, United States of America; Katholieke Universiteit Leuven, BELGIUM

## Abstract

Educators are increasingly interested in applying neuroscience findings to improve educational practice. However, their understanding of the brain often lags behind their enthusiasm for the brain. We propose that educational psychology can serve as a bridge between basic research in neuroscience and psychology on one hand and educational practice on the other. We evaluated whether taking an educational psychology course is associated with increased neuroscience literacy and reduced belief in neuromyths in a sample of South Korean pre-service teachers. The results showed that taking an educational psychology course was associated with the increased neuroscience literacy, but there was no impact on belief in neuromyths. We consider the implications of these and other findings of the study for redesigning educational psychology courses and textbooks for improving neuroscience literacy.

## Introduction

There is a growing interest in applying neuroscience findings to further educational theory, practice, and policy [1,2]. The brain is plastic–malleable in response to environmental stimuli–and educators play an important role in sculpting its structure and function through instruction. Many educators are optimistic that a better understanding of the brain will inform the design and delivery of instruction [3]. However, educators’ knowledge of the brain often lags behind their enthusiasm for the brain. They lack neuroscience literacy: an understanding of brain structure and function, of how neuroimaging techniques work, and of the scope of applying neuroscience research in educational contexts [4]. Neuroscience literacy is critical for evaluating instructional recommendations and commercial products that are purportedly based on neuroscience research. In the absence of neuroscience literacy, belief in neuromyths flourishes [5]. These are incorrect extrapolations from neuroscience findings to controversial educational ideas such as the existence of learning styles, instruction targeting the left vs. right hemispheres, the use of physical exercises for “integrating” hemispheric function during learning (i.e., Brain Gym®), and the ability of “brain games” to make people smarter [6–8].

This paper addresses two questions. The first is how best to bridge between neuroscience research and educational practice. The second is how best to improve neuroscience literacy and reduce belief in neuromyths among educators, so that they can better understand student learning and make informed evaluations of “brain-based” instruction. We propose that both questions can be answered by considering the mediating role played by educational psychology.

**Fig 1:**
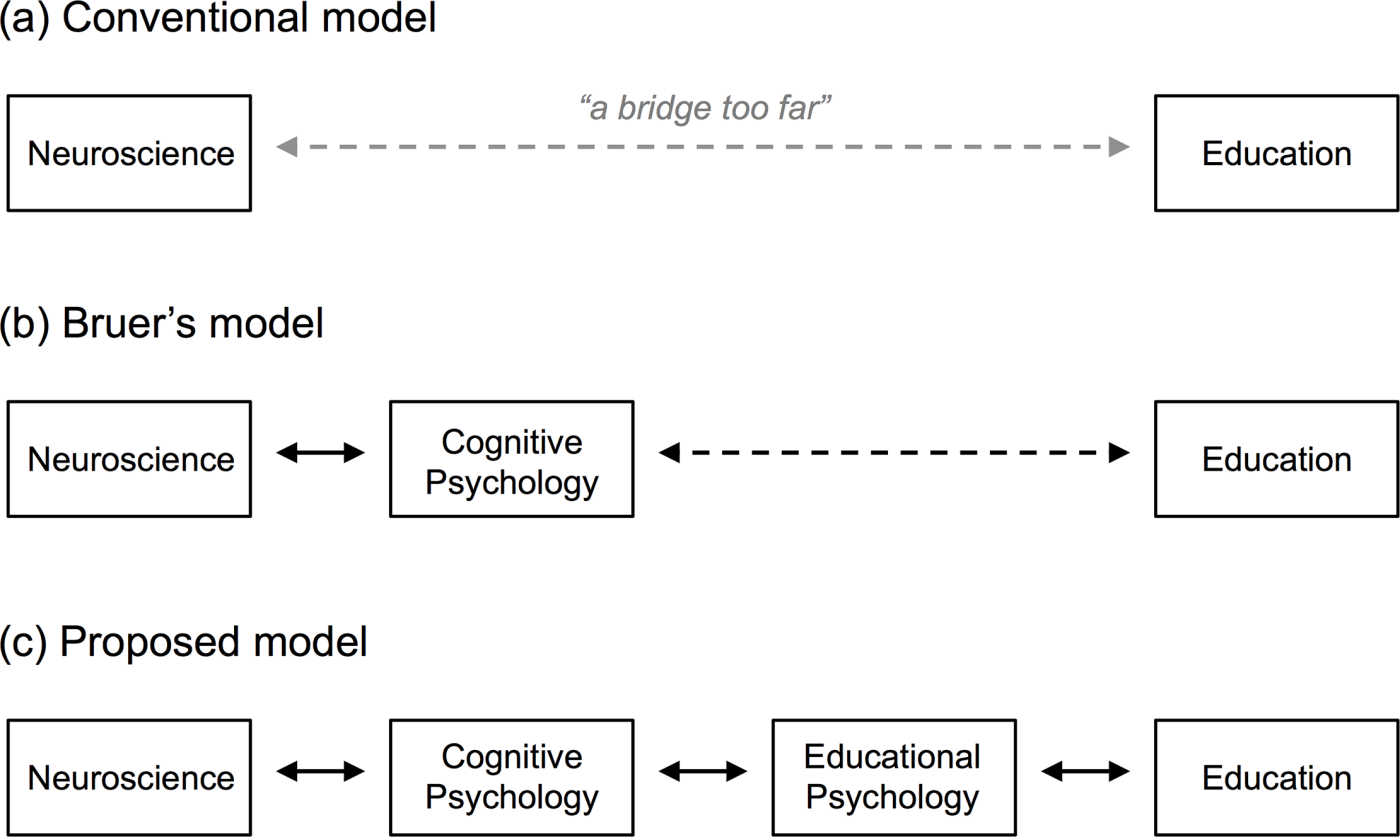
(a) Conventional model for bridging between education and neuroscience. (b) Bruer’s (1997) model. (c) Expanded model proposed here.

With respect to how best to bridge between neuroscience and education, Bruer [9] argued that the conceptual distance between the two disciplines is too far, and that this gap is one reason for the persistence of neuromyths; see Fig 1A. To solve this problem, he proposed bridging through the intermediary discipline of cognitive psychology. In this model, educational practice is grounded on the results of cognitive psychology research, which in turn is grounded on the results of neuroscience research; see Fig 1B. This model of educational neuroscience has made steady progress over the past two decades: professional societies have been organized, journals have been launched, and graduate programs have been created [10–12]. But this progress has been slow, and it is easy to spot the bottleneck: An impressive span has been erected between neuroscience and cognitive psychology, one that is traversed each day by thousands of cognitive neuroscientists [13]. By contrast, the bridge between cognitive psychology and education remains underbuilt and lightly traveled.

We propose addressing this problem by recursing: by bridging between cognitive psychology and education via the intermediate discipline of educational psychology; see Fig 1C. Educational psychology shares with education a focus on the efficacy of different instructional interventions and a commitment to implementing studies in realistic classroom settings. It shares with cognitive psychology a focus on the basic mechanisms of memory, learning, and transfer, and a methodological commitment to high levels of experimental control. As a result, it is well positioned to bridge between education and cognitive psychology, and ultimately between education and neuroscience [14].

Educational psychology is also well positioned to improve the neuroscience literacy of teachers and reduce their belief in neuromyths. Most teacher training curricula include an educational psychology course. This course covers concepts from cognitive and developmental psychology that are relevant for neuroscience. Critically, it also introduces the scientific method, which may be new to many pre-service teachers, and which is essential for understanding how to reason validly from theoretical hypotheses to experimental results, and ultimately to practical applications. Prior research has shown that taking psychology courses improves reasoning about research methods and making statistical inferences from noisy data better than taking chemistry courses [15,16]. For this reason, taking an educational psychology course might increase neuroscience literacy and reduce belief in neuromyths, even if it provides little or no direct coverage of neuroscience.

### Neuroscience literacy

We follow the OECD [17] definition of science literacy in conceptualizing neuroscience literacy as consisting of three components. *Neuroscience concepts* include the basic theoretical terminology of brain science, and the structures and functions of the brain. *Neuroscience methods* are the techniques by which neuroscientists study the brain and produce neuroscience data. *Neuroscience applications* are when neuroscientists use neuroscience findings to improve brain function and address real-world problems. Our definition of neuroscience literacy overlaps with prior definitions [4,18].

Prior studies have found that educators have low neuroscience literacy in the sense that they have difficulty interpreting neuroscience findings [8,19]. This might reflect gaps in teacher training programs, which typically do not cover the terminology of neuroscience (i.e., neuroscience concepts) nor the dominant methodologies (i.e., neuroscience methods). These gaps prevent educators from applying neuroscience findings to improve instruction and learning (i.e., neuroscience applications).

The lack of neuroscience literacy is particularly problematic because people defer to neuroscience explanations and brain images when evaluating scientific evidence [20–23]. Educators who lack neuroscience literacy are ill-equipped to evaluate neuroscience evidence offered in support of educational practices. It is therefore important to improve their neuroscience literacy to make them more discerning consumers of neuroscience research. This will help them understand when neuroscience findings are being extrapolated too far to argue for particular instructional interventions. One approach to improving the neuroscience literacy of educators is to increase the neuroscience content of pre-service and in-service teacher training [24]. Another approach is to change the information sources that educators consult to learn about neuroscience research. The current study investigated both of these approaches.

### Belief in neuromyths

Attempts to bridge between neuroscience and education have been plagued by the recalcitrant presence of neuromyths. These are widely believed–but incorrect–statements about brain structure, function, and development. Common examples include overemphasizing the importance of hemisphere dominance and the existence of critical periods during early development [8]. Some neuromyths have their origins in the positive intentions of educators eager to translate new findings about learning and plasticity into new instructional approaches, and of policy makers searching for neuroscience findings to support their decisions [8,25]. Other neuromyths have more dubious origins in the marketing campaigns of commercial publishers of curricular materials eager to exploit educators’ interest in neuroscience [26].

Regardless of their origins, neuromyths are widespread and have proven difficult to dispel. For example, many educators (and members of the public) believe that the left hemisphere is logical, associated with language and analytic thinking, whereas the right hemisphere is intuitive, associated with spatial reasoning and creativity. This neuromyth likely has its origins in misunderstandings of seminal neuroscience studies of the cognitive deficits of split-brain patients [27,28]. In fact, contemporary neuroimaging studies have revealed that language understanding recruits a network of areas that spans both hemispheres [29]. Nevertheless, the early split-brain research led to the development of “left vs. right brain” curricula that segregated reading and writing from working with visual representations [5]. At best, these lessons are no better than conventional instruction; at worst, they may undermine children’s attempts to integrate linguistic and spatial thinking [30].

There are many other prevalent neuromyths. These include an overemphasis on early interventions using “enriched environments”; that people only use 10% of their brains; that there exist multiple intelligences or learning styles, each with a separate neural correlate; and that physical exercises can improve communication between the left and right hemispheres [5,9]. These neuromyths have led to instructional and policy recommendations that ultimately waste time, money, and effort [31]. It is therefore important to reduce belief in neuromyths among educators, to make them more critical consumers of neuroscience findings as reported by public media and as utilized by commercial interests and policy makers.

The current study investigates whether taking an educational psychology course is associated with reduced belief in neuromyths. This might be because educational psychology includes concepts from cognitive and developmental psychology that are critical for understanding the results of research in cognitive and developmental neuroscience. This might also be because educational psychology courses typically cover the scientific method, which may be new to many pre-service teachers. An understanding of the scientific method might transfer to better understanding and evaluation of neuroscience studies. As noted above, there is evidence that taking psychology courses improves reasoning about research methods and inferences from data [15,16].

### Measuring neuroscience literacy and belief in neuromyths

There have been several prior attempts to measure neuroscience literacy and belief in neuromyths. Herculano-Houzel [32] constructed a survey and administered it to members of the Brazilian public. Greater neuroscience literacy was associated with reading popular science magazines and newspapers. Greater neuroscience literacy was also modulated by prior schooling, with people with graduate degrees scoring 30% higher than people with high school degrees. Nevertheless, neuromyths were widespread across all levels of educational attainment. For example, 59% of participants with college degrees believed the neuromyth that “We use only 10% of the brain.” Other widespread neuromyths concerned the relationship between emotion and reasoning, the brain-as-computer metaphor, and the effect of physical exercise on brain activity.

Howard-Jones et al. [19] adapted the Herculano-Houzel [32] survey and administered it to a sample of pre-service teachers in the UK. More than 50% of pre-service teachers believed neuromyths about enriched environments (89%), learning styles (82%), hemispheric dominance (60%), and the benefits of physical exercise on the integration of hemispheric function (65%). In addition, greater neuroscience literacy was associated with reduced belief in neuromyths.

Dekker et al. [6] compared the neuromyths believed by in-service teachers in the UK and the Netherlands. Their survey combined items from Howard-Jones et al. [19] and the OECD [8] report. More than 80% of in-service teachers believed neuromyths about learning styles, hemispheric dominance, and the benefits of physical exercises on the integration of hemispheric function. In addition, 48% of UK teachers and 46% of Netherlands teachers believed the pesky neuromyth that we only use 10% of our brain. Neuroscience literacy was positively associated with reading popular science magazines, consistent with Herculano-Houzel [32]. A surprising finding was that greater neuroscience literacy was associated with *increased* belief in neuromyths, a result that runs counter to Howard-Jones et al. [19].

These findings have been replicated and extended in more recent studies using larger samples and more diverse participants, including members of the general public and educators in Latin America, Europe, and the United States [33–37]. Of particular note, educators believe neuromyths about the singular importance of enriched early environments, the psychological reality of learning styles, and learning benefits of Brain Gym®. Higher levels of educational attainment, more coursework in neuroscience and the biological sciences, and more frequent consultation of reliable information sources such as scientific journals are generally associated with increased neuroscience literacy and reduced belief in neuromyths–although there are some contradictory findings, a point we return to below when discussing the results of the current study. More generally, these studies are important because they demonstrate that surveys can be used to measure neuroscience literacy and belief in neuromyths.

### The current study

The current study investigated the potential of educational psychology to bridge between education and neuroscience. Specifically, it evaluated whether taking an educational psychology course is associated with increased neuroscience literacy and reduced belief in neuromyths in pre-service teachers. Although conventional educational psychology courses do not provide much coverage of neuroscience, they do present concepts from cognitive and developmental psychology for interpreting the findings of cognitive and developmental neuroscience studies. They also emphasize the scientific method, research methods, and reasoning from theory and data to applications–skills that might transfer to the evaluation of neuroscience research [15,16].

There were three research questions: (1) Is taking an educational psychology course associated with increased neuroscience literacy? (2) Is taking an educational psychology course associated with decreased belief in neuromyths? (3) Do factors such as (i) the information sources consulted to learn about neuroscience findings and (ii) prior coursework in the biological sciences modulate neuroscience literacy and belief in neuromyths in pre-service teachers?

To address these research questions, we focused on a sample of South Korean pre-service teachers majoring in elementary education. We chose this population for two reasons. First, neuroscience literacy and belief in neuromyths have been comparatively understudied in Asian countries [38], with only one prior study focusing on Korea [39]. Second, elementary education pre-service teachers are interesting because they generally have fewer opportunities to take science courses than their secondary education counterparts, who can specialize in science or mathematics education. We measured neuroscience literacy and belief in neuromyths using a survey that extended prior surveys [6,19,32]. We administered the survey at the beginning and end of the semester, to measure the impact of taking an educational psychology course. We also collected data about the information sources consulted to learn about neuroscience findings and about prior coursework in the biological sciences because these have previously been found to modulate neuroscience literacy and belief in neuromyths [6,32].

## Materials and methods

### Participants

Participants were recruited from a public university in Seoul, South Korea. All were sophomore pre-service teachers in the elementary teacher training program. To earn an elementary teaching certification in South Korea, pre-service teachers must complete the required teacher training curriculum at one of 13 government-designated universities for elementary education. The experimental group consisted of pre-service teachers taking the required educational psychology course, and were tested during the Spring 2014 semester. The control group consisted of pre-service teachers who had completed the same coursework as the experimental group but who had not taken and were not enrolled in the required educational psychology course; they were tested during the Spring 2017 semester. Participants in both groups completed the survey twice, once at the beginning of the semester and once at the end. The experimental group consisted of 50 participants (35 female, *M* age = 20.86 years, *SD* = 2.33) and the control group consisted of 49 participants (37 female, *M* age = 20.27 years, *SD* = 1.15). They received course credit for their participation.

### Materials

#### Neuroscience knowledge survey

We developed a neuroscience knowledge survey; see S1 Appendix. It consists of two parts. The first part measures neuroscience literacy and belief in neuromyths. Of the 60 items, 47 items were adapted from prior surveys of neuroscience literacy and belief in neuromyths [6,32,40–47]. Most items came from the Herculano-Houzel [32] and Dekker et al. [6] surveys. We intentionally omitted items testing belief in neuromyths about learning styles and multiple intelligences, which have been used in prior research. These proposals originated in psychology [48,49], and thus belief in them might reflect misconceptions about this discipline rather than neuroscience. We wrote 13 additional items about neuroscience research on topics such as synaptogenesis, myelination, glia cells, emotional processing (i.e., affective neuroscience), the use of cognitive enhancers, and neuroimaging methodologies by consulting recent introductory neuroscience textbooks.

The determination of whether an item was correct or incorrect was made by five faculty members in departments of neuroscience, educational psychology, science education, and elementary education. There were disagreements about two items: “The volume of blood in the brain increases with physical effort” and “Blind people have better hearing.” Most–but not all–faculty members judged these to be incorrect. This disagreement was resolved by further discussion and by consulting the recent literature. The former statement was ultimately judged to be incorrect because of research demonstrating that the cerebral autoregulation mechanism preserves the volume of blood in the brain [50]. The latter statement was also judged to be incorrect and included in the survey. However, one of the reviewers prompted us to reconsider this judgment given research on the enhanced ability of blind people to localize sounds in space [51,52]. We therefore omitted this item, and all analyses below considered only 59 items.

Items were organized into six sections, each representing a different domain of interest. Section *I*. *General knowledge* contained 13 items (3 correct and 10 incorrect) about the neural correlates of intelligence, memory, dreaming, music, and bilingualism. Section *II*. *Brain function* contained 8 items (3 correct and 5 incorrect) about the localization and lateralization of brain function. Section *III*. *Brain development* contained 10 items (7 correct and 3 incorrect) about brain development in young children and adolescents. Section *IV*. *Brain structure* contained 12 items (7 correct and 5 incorrect) about basic brain anatomy and physiology. Section *V*. *Neuroimaging* contained 6 items (3 correct and 3 incorrect) about what neuroimaging measures reflect and their valid interpretation. Finally, section *VI*. *Applying neuroscience results* contained 10 items (5 correct and 5 incorrect) about applying neuroscience findings to reason about drugs, diet, and diseases of the brain. Within each section, correct and incorrect statements were randomly ordered.

Participants judged each statement as correct (“Yes”), incorrect (“No”), or responded “I don’t know.” The number of correct answers across all 59 items constituted the neuroscience literacy score (Cronbach’s alphas > .721 for the pre- and post-test administrations for the experimental and control groups). We defined a subset of the items as neuromyths items according to two criteria, as described below in the Results section. The number of incorrect answers across the neuromyth items constituted the belief in neuromyths score.

The survey also collected background information about participants. This included their age, gender (with options “male” and “female”), number of biological sciences courses taken (“none”, “one”, “two”, and “three or more”), and information sources consulted to learn about neuroscience research (“no sources”, “books”, “commercial products”, “pre-service training”, “public media”, “scientific journals”, and “other”). Age had an open response format and all other items had a multiple-choice format. For the information sources item, multiple options could be selected.

The survey was created in English and translated into Korean. The accuracy of translation was confirmed by double-blind peer review.

#### Educational psychology course

The goal of the educational psychology course is to present theories and evidence from psychology relevant for education to pre-service teachers. The instructor was a professor trained in education and cognitive psychology. The course materials included textbook readings, lectures, assignments, and exams. The textbook was a Korean translation of Eggen and Kauchak’s [53] *Educational Psychology*: *Windows on classrooms* (*9*^*th*^ *edition*). This textbook covers cognitive, social, and emotional development; learning, memory, and complex cognition; behaviorism; motivation; instruction; individual differences and intelligence; exceptional learners; classroom management; assessment; and standardized testing.

The neuroscience-relevant content of the course included a section of the textbook and the instructor’s lecture notes. The textbook chapter on cognitive and language development describes the structure of neurons, the function of the cerebral cortex, the nature of myelination, and the controversy surrounding “brain-based learning.” In addition, it debunks two neuromyths that were measured by the neuroscience knowledge survey, about hemispheric differences and critical periods. The instructor lecture notes introduced the structure of brain stem, the limbic system, and the cerebral cortex, and also specify how information is transmitted in the brain. Overall, the educational psychology course was conventional in structure.

### Procedure

The study was approved by the Institutional Review Board at the University of Minnesota (#1306P37021). The pre-test was administered at the beginning of the semester. The consent form explained that the purpose of the study was to investigate educators’ understanding of neuroscience research related to learning and instruction. In particular, the terms “neuroscience literacy” and “neuromyths” were not used. Participants provided informed consent and then completed both parts of the neuroscience knowledge survey.

Participants were not informed that there would be post-test at the end of semester to ensure that they did not engage in extra-curricular study of neuroscience. The instructor was not shown the neuroscience knowledge survey during the semester to ensure he did not tailor his instruction to its contents.

The post-test was administered three months later, at the end of the semester. The items were same at post-test as at pre-test, but their order was changed within each subsection. For the participants in the experimental group, the post-test was introduced as an assessment tool for evaluating the impact of taking an educational psychology course on their understanding of neuroscience relevant to education. For participants in the control group, it was introduced as an assessment tool for evaluating the impact of their overall program during the semester. Again, the terms “neuroscience literacy” and “neuromyths” were not used. Participants provided informed consent and completed just the first part of the survey. (The second part was not administered because it was assumed that their background information had not changed.)

## Results

### Neuroscience literacy

**Fig 2:**
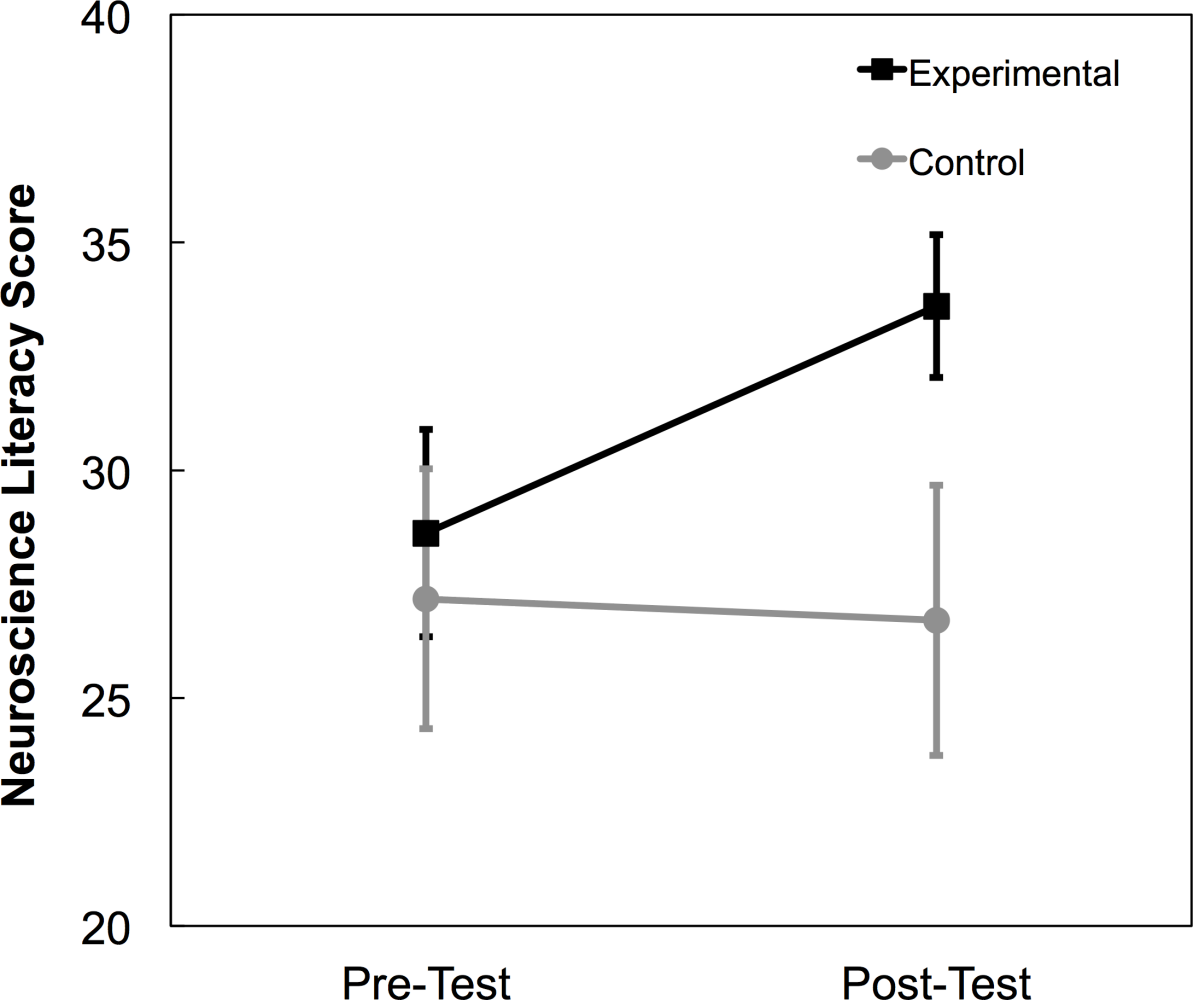
Neuroscience literacy results. Pre-service teachers in the experimental group had greater overall neuroscience literacy scores at post-test than their counterparts in the control group. Error bars represent 95% CIs.

The first research question is whether taking an educational psychology course is associated with increased neuroscience literacy. To answer this question, we analyzed neuroscience literacy scores in a two-way repeated measures ANOVA with between-subjects factor group (experimental, control) and within-subjects factor time (pre, post). There were main effects of group (*F*(1, 97) = 6.680, *p* = .011, *η*_*p*_^*2*^ = .064) and time (*F*(1, 97) = 8.152, *p* = .005, *η*_*p*_^*2*^ = .078). Critically, these were qualified by a significant interaction (*F*(1, 97) = 11.898, *p* = .001, *η*_*p*_^*2*^ = .109), shown in Fig 2. Breaking down this interaction, at pre-test, there was no difference in the neuroscience literacy scores of experimental group (*M* = 28.62, *SD* = 8.2) and the control group (*M* = 27.18, *SD* = 10.1) (*p* = .440). However, at post-test, the experimental group (*M* = 33.60, *SD* = 6.1) had significantly higher scores than the control group (*M* = 26.71, *SD* = 10.6) (*t*(76.393) = 3.954, *p* < 001, *d* = 0.80). This provides evidence that taking an educational psychology course improves pre-service teachers’ neuroscience literacy.

To better understand this finding, we analyzed the pre-post improvement of each group on each of the six sections (see Table 1). The experimental group showed pre-post gains on five of the six sections (*t*s(49) > 2.25, *p*s < .047, *d*s > 0.33), with the largest effect for section *III*. *Brain development* (*d* = 0.81). The only section that showed no improvement was section *I*. *General knowledge*, although the trend was in the predicted direction. By contrast the control group showed no pre-post gains on any of the six sections (*p*s > .152).

**Table 1:** Neuroscience literacy scores for the two groups at the two time points.

	Pre		Post				
Section (Number of items)	*M*	*SD*	*M*	*SD*	*t*	*p*	*d*
Experimental group (*N* = 50)							
I. General knowledge (13)	7.18	2.4	7.82	2.1	1.90	.063	0.29
II. Brain function (8)	3.54	1.4	4.14	1.1	3.00	.004	0.49
III. Brain development (10)	4.42	2.2	6.00	1.7	6.30	< .001	0.81
IV. Brain structure (12)	5.42	2.0	6.04	1.8	2.25	.029	0.33
V. Neuroimaging (6)	2.48	1.6	3.46	1.4	3.85	< .001	0.66
VI. Applying neuroscience (10)	5.58	1.6	6.14	1.8	2.04	.047	0.33
Total (59)	28.62	8.2	33.60	6.1	5.65	< .001	0.69
Control group (*N* = 49)							
I. General knowledge (13)	7.29	2.4	7.20	2.4	-0.26	.793	-0.04
II. Brain function (8)	3.16	1.5	3.24	1.6	0.31	.761	0.05
III. Brain development (10)	4.59	2.5	4.69	2.4	0.28	.784	0.04
IV. Brain structure (12)	4.27	2.5	4.00	2.7	-0.64	.525	-0.10
V. Neuroimaging (6)	2.39	1.7	2.55	1.7	0.65	.519	0.10
VI. Applying neuroscience (10)	5.49	2.4	5.02	2.5	-1.46	.152	-0.19
Total (59)	27.18	10.2	26.71	10.6	-0.36	.723	-0.05

### Belief in neuromyths

The second research question is whether taking an educational psychology course is associated with decreased belief in neuromyths. To address this question, we defined neuromyths in two ways. The first definition was operational, as incorrect items that were answered incorrectly (i.e., received a “yes” response) by at least 50% of the experimental or control participants at pre-test. Note that larger scores indicate greater belief in neuromyths. This definition was inspired by prior research on psychological misconceptions [54,55]. By this definition, there were 8 neuromyths (see Table 2). Six were common to both two groups. These included classic neuromyths such as over-emphasizing early interventions using “enriched environments” (item III-6). These also included neuromyths promoted by commercial programs about the utility of physical exercise for improving communication between the left and right hemispheres (item II-6). Of the two neuromyths that were particular to the experimental group, item VI-4 was believed by 49% of control participants, just below the 50% threshold.

**Table 2:** Operationally defined neuromyths believed by 50% or more of participants in either group at pre-test.

	Experimental		Control	
Neuromyth	Pre	Post	Pre	Post
II-4. *Right-hemisphere learners are more creative than left-hemisphere learners*.	64	62	53	37
II-6. *Brief co-ordination exercises can improve integration of left and right hemispheric brain function*.	70	86	57	65
II-7. *The left side of the brain deals with rational thinking and the right side is emotional processing*.	82	80	55	59
III-6. *Environments that provide rich stimuli improve the brain function of pre-school children*.	88	98	84	90
III-9. *There are critical periods in childhood after which certain abilities can no longer develop and certain skills can no longer be learned*.	66	88	55	59
IV-3. *The volume of blood in the brain increases with physical effort*.	74	86	76	63
VI-4. *Children are less attentive after consuming sugary drinks and/or snacks*.	64	54	49	31
VI-6. *It has been scientifically proven that fatty acid supplements (omega-3 and omega-6) have a positive effect on academic achievement*.	60	46	37	24

The data are the percentage of participants that incorrectly endorsed the neuromyth (i.e., responded “yes” to an incorrect statement).

**Fig 3:**
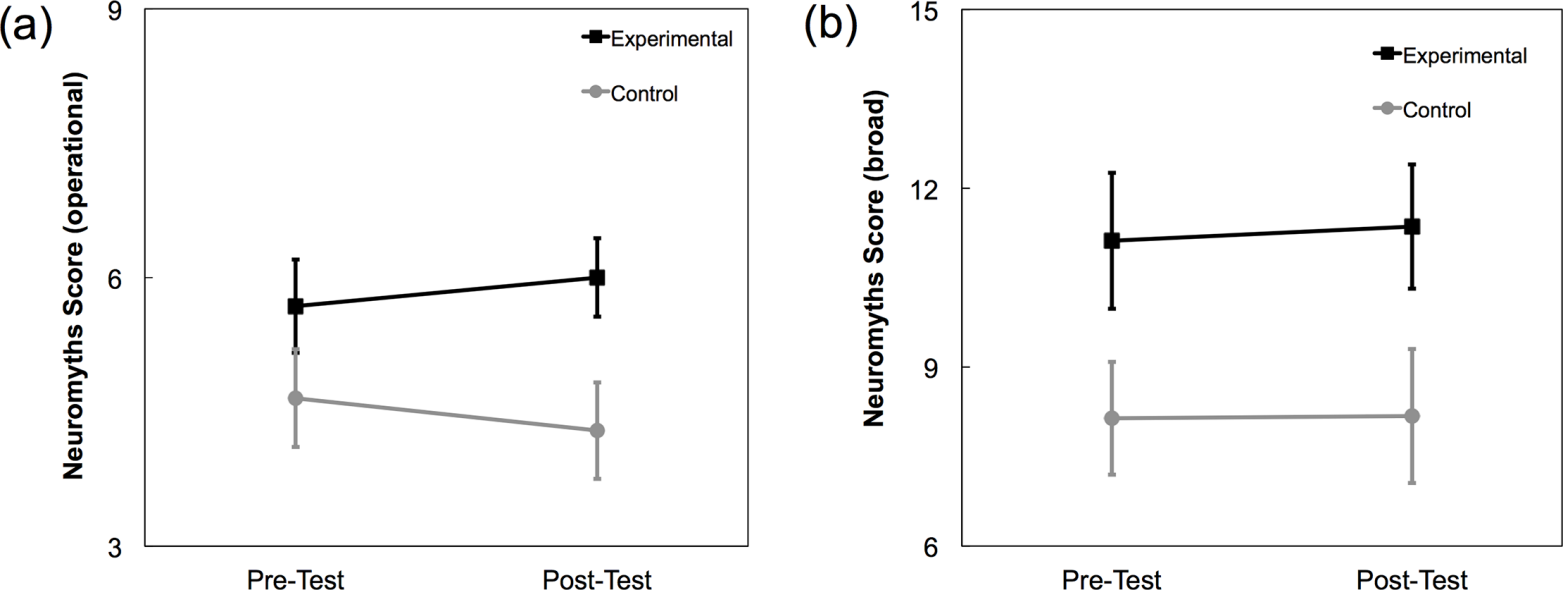
Belief in neuromyths results. Neuromyths were defined (a) operationally (*max* = 8) and (b) broadly (*max* = 31). In both cases, belief in neuromyths among participants in the experimental group was unchanged after taking an educational psychology course. Error bars represent 95% CIs.

Given this operational definition, we analyzed whether taking an educational psychology course reduced belief in neuromyths. A two-way repeated measures ANOVA with between-subjects factor group (experimental, control) and within-subjects factor time (pre, post) revealed a main effect of group (*F*(1, 97) = 19.319, *p* < .001, *η*_*p*_^*2*^ = .166), no main effect of time (*p* = .905), and no interaction of group and time (*p* = .087); see Fig 3A. The main effect of group indicates that participants in the experimental group (*M* = 5.84, *SD* = 1.7) believed more neuromyths than participants in the control group (*M* = 4.47, *SD* = 1.9), and the absence of an interaction indicates that taking an educational psychology course does not close this gap.

A potential limitation of defining neuromyths to be the most recalcitrant incorrect statements is that this may have stacked the deck against finding an effect of taking an educational psychology course. We therefore considered a broader definition of a neuromyth as any incorrect statement in the survey, and computed the number of the 31 incorrect items that were answered incorrectly (i.e., received a “yes” response). This broader definition makes sense because most of the incorrect items were taken from prior studies of belief in neuromyths, specifically the Herculano-Houzel [32] study of the general public and the Dekker et al. [6] study of in-service teachers. We adopted the same analysis strategy as above: a two-way repeated measures ANOVA with between-subjects factor group (experimental, control) and within-subjects factor time (pre, post). Again, there was a main effect of group (*F*(1, 97) = 21.083, *p* < .001, *η*_*p*_^*2*^ = .179), with participants in the experimental group (*M* = 11.24, *SD* = 3.9) believing more neuromyths than their counterparts in the control group (*M* = 8.16, *SD* = 3.7), and again neither the main effect of time (*p* = .712) nor the interaction of group and time (*p* = .793) reached significance; see Fig 3B. Thus, the prediction that taking an educational psychology course is associated with decreased belief in neuromyths was not supported regardless of whether neuromyths were defined operationally (and narrowly) or whether they were defined broadly.

### Modulating factors

The third research question concerned factors that modulate neuroscience literacy and belief in neuromyths. We first investigated the information sources that participants reported consulting to learn about neuroscience findings (see Table 3). We did not expect differences between the groups because all participants were sampled from the same population (i.e., pre-service teachers at the same institution) and responses were recorded at pre-test, before the experimental group experienced an educational psychology course. As expected, a series of *z*-tests found that no difference in the proportion of each group that consulted each information source (*ps* > .322), with the exception of commercial products, which a greater proportion of the experimental group reported consulting (*p* = .043). Across all participants, the most frequently consulted information sources were public media (*N* = 53, 53.5%), books (*N* = 32, 32.3%), and “no sources” (*N* = 15, 15.2%).

**Table 3:** Information sources consulted to learn about neuroscience research by experimental and control pre-service teachers.

	Experimental (*N* = 50)		Control (*N* = 49)				
Source	*N*	%	*N*	%	*z*		*p*
Public media	25	50.0	28	57.1	-0.708		.478
Books	14	28.0	18	36.7	-0.926		.352
Scientific journals	8	16.0	5	10.2	0.854		.395
Commercial products	4	8.0	0	0	2.021		.043
Pre-service training	1	2.0	0	0	0.995		.322
Other	3	6.0	3	6.1	-0.021		.984
No sources	9	18.0	6	12.2	0.805		.418

Because selection of multiple options was allowed, the percentages sum to more than 100% across the seven sources.

We analyzed whether particular information sources were associated with higher neuroscience literacy or lower belief in neuromyths. This analysis was complicated by the fact that participants could select multiple information sources and by the fact that some information sources were selected by very few participants. We therefore constructed a between-subjects factor, information source, with four levels: only public media, only books, “no sources”, and multiple sources. Eighty-eight participants could be assigned to one of these four levels. We conducted one-way ANOVAs with information source as the factor on each of three dependent measures: neuroscience literacy, belief in neuromyths defined operationally, and belief in neuromyths defined broadly. These were the measures collected at pre-test, before the experimental group experienced the educational psychology course. There was no effect of information source on any of the dependent measures (*p*s > .05); see Table 4. These findings suggest that the information sources pre-service teachers consult do not modulate their neuroscience literacy or belief in neuromyths.

**Table 4:** One-way ANOVAs of consulted information sources on the outcomes of neuroscience literacy and belief in neuromyths at pre-test.

		Neuroscience Literacy		Neuromyths (Operational)		Neuromyths (Broad)	
Source	*N*	*M*	*SD*	*M*	*SD*	*M*	*SD*
Public media	38	26.76	7.9	5.16	1.7	9.29	3.1
Books	16	30.69	8.7	5.63	1.9	10.56	4.8
No sources	15	26.80	11.7	4.20	1.9	8.27	3.7
Multiple sources	19	29.95	8.9	5.89	2.0	11.11	4.7
*F*(3, 84), *p*		1.101, 0.354		2.634, 0.055		1.910, 0.134	

The other potential modulating factor was number of biological sciences courses taken. Unfortunately, the teacher training curriculum is highly prescribed in South Korea, with no opportunity to take such courses. Moreover, only 8% of all participants reported taking biological sciences courses at previously attended institutions. For these reasons, we could not investigate whether taking biological sciences courses modulated neuroscience literacy or belief in neuromyths.

## Discussion

It is important for educators and the general public to be intelligent consumers of the neuroscience findings increasingly used to argue for new educational programs [13,56]. Prior studies have *measured* neuroscience literacy and belief in neuromyths among pre-service teachers, in-service teachers, and the general public [6,19,32]. The current study took the further step of investigating whether neuroscience literacy and belief in neuromyths *change* after taking an educational psychology course, focusing on pre-service teachers. The study found support for this hypothesis–and also identified its limits. Taking an educational psychology course improved neuroscience literacy, but did not reduce belief in neuromyths. These findings were *not* modulated by the information sources participants reported consulting. Here, we consider the implications of these results, identify limitations of the current study, and explore directions for future research.

### Neuroscience literacy

Taking an educational psychology course was associated with increased neuroscience literacy in pre-service teachers. To understand this finding, we return to the three components of neuroscience literacy developed above–neuroscience concepts, neuroscience methods, and neuroscience applications–and consider how taking an educational psychology course might improve each one.

Neuroscience concepts include the basic terminology of brain science, and the structures and functions of the brain. Educational psychology textbooks provide direct coverage of foundational neuroscience concepts. They describe the basic structure and function of the brain, i.e., what neurons are, how they are connected by synapses, and how the strength of synaptic connections changes through long-term potentiation. They also describe how the brain changes over development, explaining concepts such as synaptogenesis and plasticity. This was true of the textbook used by the pre-service teachers in this study, and it is true of competing textbooks in the marketplace. Thus it is perhaps not surprising that the largest gain in neuroscience literacy for participants in the experimental group was for the section of *III*. *Brain development*. The implication is that improving course coverage of the neuroscience concepts most relevant for education–including the neural correlates of individual differences in mathematical, reading, and science ability–may improve the neuroscience literacy of pre-service teachers.

Neuroscience methods include the techniques by which neuroscientists measure brain structure and function, and ultimately produce neuroscience data. Educational psychology textbooks provide relatively little coverage of neuroscience methods. Nevertheless, taking an educational psychology course was associated with better performance on section *V*. *Neuroimaging*. To understand this improvement, consider that educational psychology is an important component of teacher training in part because it provides a *scientific* understanding cognition, learning, development, and other topics relevant to education [57–59]. The first chapter of textbooks typically introduces the scientific method, explains the difference between correlational versus experimental designs, and discusses the statistical interpretation of data. Later chapters on assessment and standardized testing cover the valid measurement and interpretation of student data. Prior research has shown that taking psychology courses improves general reasoning about scientific research methods and noisy data [15,16]. For this reason, we interpret the finding of improved reasoning about neuroimaging data after taking an educational psychology course as an example of positive transfer.

Neuroscience applications use neuroscience findings to improve brain function and address real-world problems. Educational psychology textbooks sequester their coverage of neuroscience from their coverage of psychological and educational topics. As a result, they provide little support for applying neuroscience findings to understand whether instructional interventions will work, and if so, why. Nevertheless, taking an educational psychology course was associated with increased knowledge of section *VI*. *Applying neuroscience results*. We again interpret this improvement as an example of positive transfer. Educational psychology courses provide numerous examples of applying the results of psychological (i.e., behavioral) studies conducted in the laboratory to improve student learning in the classroom. These might serve as models for valid inferences about applying neuroscience findings to educational practice.

### Belief in neuromyths

Contemporary educational psychology textbooks discuss and debunk neuromyths. For example, the textbook used in the current study explicitly addressed neuromyths about hemispheric differences and critical periods [53]. Nevertheless, taking an educational psychology course did *not* reduce belief in neuromyths. This was surprising. One reason may be because educational psychology is just one of several bridging disciplines between education and neuroscience [9,60]. Improving knowledge of one bridging discipline may not be sufficient for dispelling neuromyths. Broader instruction in cognitive psychology, developmental psychology, statistics, and experimental design may be necessary.

A broader consideration of the literature suggests that the neuromyths that people believe may be changing over time. In particular, we propose two classes of neuromyths. *Stable* neuromyths are incorrect beliefs that continue to be widespread today, even in the face of disconfirming evidence. Examples include the neuromyth about exaggerated differences between the left and right hemispheres and the neuromyth about the effectiveness of physical exercises on the integration of hemispheric function. These neuromyths have been documented by prior studies [6,38], and were believed by more than 50% of the pre-service teachers in our sample. *Declining* neuromyths are incorrect beliefs that were widespread in the past but that are becoming less prevalent. For example, a neuromyth that was once widely believed is that exposing infants to classical music increases their educational achievement later in life (i.e., the “Mozart effect”). However, this neuromyth was believed by less than 50% of pre-service teachers in our sample.

That belief in some neuromyths appears to be declining suggests that, over time, carefully communicated neuroscience findings can supersede incorrect beliefs. Indeed, Macdonald et al. [35] found that younger people believe neuromyths less strongly than older people. Declining belief in neuromyths *may* also explain the surprising finding that participants in the experimental group believed more neuromyths than participants in the control group. This is because the control group data were collected three years after the experimental group data, and the difference may reflect a general decline in belief in some neuromyths (see Table 2). That other neuromyths are persistent indicates that the job is far from done [61,62].

### Modulating factors

There was no relationship between the information sources participants reported consulting and their neuroscience literacy or their belief in neuromyths. This null finding adds to a mixed literature on modulating factors. It is inconsistent with prior studies finding that reading books and science magazines is associated with increased neuroscience literacy [6] and reduced belief in neuromyths [33] among in-service teachers. However, it is consistent with prior studies finding no such association among pre-service teachers [19,36]. These mixed findings might reflect differences in the instruments used and differences in the demographics of pre-service and in-service teachers across countries. Future research is needed to resolve these differences, and to investigate additional factors that modulate neuroscience literacy and belief in neuromyths among educators.

### Limitations and future directions

There are at least three limitations of the current study. The first is that the design does not support inferences about which components of the educational psychology course were responsible for increasing neuroscience literacy. Our explanation highlighted two components. The first is that educational psychology courses cover scientific research methods and the statistical interpretation of data, and this knowledge might transfer to evaluating the findings of neuroscience studies and their application to educational problems [15,16]. The second is that educational psychology courses provide examples of how laboratory studies of psychological (i.e., behavioral) phenomena can be applied to improve educational practice, and these might guide the application of neuroscience findings. Future studies should investigate whether these components–or others such as reviewing foundational neuroscience concepts or delineating the development of cognition–drive the current findings. These studies might, for example, measure neuroscience literacy after each chapter rather than just at the beginning and end of the semester.

A second limitation stems from using an educational psychology course that was conventional in content and structure. The disadvantage of using such a course is that it does not fully exploit the expanded model of educational neuroscience and teacher training shown in Fig 1C. This model proposes that educational psychology is critical for bridging between the lower-level constructs of neuroscience and the higher-level constructs of cognitive and developmental psychology, and ultimately for guiding neuroscience applications to educational problems. To better test the expanded model, future studies should design and evaluate innovative educational psychology courses that do not sequester neuroscience concepts and methods in an early section that is never referenced again, but rather interleave neuroscience, psychological, and educational content throughout. The prediction is that such an integrated course will produce larger increases in neuroscience literacy and statistically significant decreases in belief in neuromyths.

The third limitation concerns the generalizability of the current results to other populations. We sampled a small number of pre-service teachers majoring in elementary education in South Korea. An advantage of this sample is that Asian countries have been understudied in the neuroscience literacy and neuromyths literature [6,38,39]. A disadvantage is that it is unclear whether taking an educational psychology course improves the neuroscience literacy of other groups: pre-service teachers majoring in other areas (e.g., science education at the secondary level), in-service teachers, and educators from other countries. Future studies should reduce this uncertainty by studying other populations. Doing so will bring two additional benefits. First, it will contribute to the growing literature on cross-cultural differences in neuroscience literacy and belief in neuromyths among educators [6,33,34,38]. Second, it may support inferences about the factors that modulate neuroscience literacy and belief in neuromyths. The current study was limited in this regard in part because of the homogeneity of the sample.

## Conclusion

Prior researchers have proposed bridging between neuroscience and education through the intermediary field of cognitive psychology [9,13,60], as shown in Fig 1B. We argued that progress has been slowed by the distance between cognitive psychology and education, and proposed bridging these two disciplines through the intermediary discipline of educational psychology, as shown in Fig 1C. As tentative evidence for the efficacy of this proposal, taking an educational psychology course was associated with increased neuroscience literacy in pre-service teachers, although belief in neuromyths remained unchanged. Future studies should further investigate the potential–and limits–of teacher training programs for strengthening the connection between neuroscience and education [1].

## Supporting information

S1 Appendix Neuroscience knowledge survey. (DOCX)

S1 Dataset Overall data. (TXT)

S2 Dataset Original survey response data. (XLSX)
