# Supplementary material for: Taking an educational psychology course improves neuroscience literacy but does not reduce belief in neuromyths
Source: PLoS One. 2018 Feb 5;13(2):e0192163. doi: 10.1371/journal.pone.0192163 (PMC5798820; doi:10.1371/journal.pone.0192163)
Supplement: S1 Appendix — (DOCX) [file pone.0192163.s001.docx]

Supporting information

Taking an educational psychology course improves neuroscience literacy

but does not reduce belief in neuromyths

Soo-hyun Im^1^, Joo-Yun Cho^2^, Janet M. Dubinsky^3^, and Sashank Varma^1^

**Affiliations:**

^1^ Department of Educational Psychology, University of Minnesota, Minneapolis, MN, USA

^2^ Department of Elementary Education, Seoul National University of Education, Seoul, South Korea

^3^ Department of Neuroscience, University of Minnesota, Minneapolis, MN, USA

* Corresponding author

E-mail: [imxxx045@umn.edu](mailto:imxxx045@umn.edu) (SI)

**S1 Appendix. Neuroscience knowledge survey**

I. General knowledge (14 statements)

II. Brain function (8 statements)

III. Brain development (10 statements)

IV. Brain structure (12 statements)

V. Neuroimaging (6 statements)

VI. Applying neuroscience results (10 statements)

Incorrect items are shown in italics.

Item I-4 is shown underlined. This item was originally judged to be incorrect. However, one of the anonymous reviewers pointed out that there is in fact mixed evidence in the literature for this item. For this reason, we did not include it in the final analysis.

Percentages which changed significantly from pre-test to post-test are shown in bold (McNemar-Bowker test, *p* < .05 level).

A number of items were adapted from prior studies:

- Herculano-Houzel (2002): I-3, I-6, I-7, I-13, I-14, II-2, III-1, IV-2, IV-3, IV-8, IV-9, IV-12, V-2, VI-1, VI-3, VI-9, and VI-10;
- Dekker et al. (2012): I-1, I-2, I-11, II-6, III-2, III-4, III-6, III-9, III-10, VI-4, VI-5, and VI-6;
- MacNabb et al. (2000): I-10, II-1, II-4, III-5, and IV-4;
- Tokuhama-Espinosa (2010): I-10, II-1, II-4, III-5, and IV-4;
- Howard-Jones (2007): IV-6 and IV-10;
- Marcus (2012): V-1 and V-4;
- Schultz (2009): II-7;
- Simons and Chabris (2012): I-12;
- GSLC (2011): IV-11
- Beck (2010): V-3;

The remaining items were created for the current study.

(Survey follows on the next page.)

***Instructions: Please indicate your agreement with each of the following statements by marking “Yes”, “No”, or “Don’t Know”.***

|  | Experimental Group | | | | | | | |  | Control Group | | | | | | | |
| --- | --- | --- | --- | --- | --- | --- | --- | --- | --- | --- | --- | --- | --- | --- | --- | --- | --- |
|  | Yes (%) | |  | No (%) | |  | I don’t know (%) | |  | Yes (%) | |  | No (%) | |  | I don’t know (%) | |
|  | Pre | Post |  | Pre | Post |  | Pre | Post |  | Pre | Post |  | Pre | Post |  | Pre | Post |
| **I. General knowledge (14 statements)** |  |  |  |  |  |  |  |  |  |  |  |  |  |  |  |  |  |
| *1. We only use 10% of our brain.* | 34 | 36 |  | 38 | 42 |  | 28 | 22 |  | 47 | 35 |  | 24 | 33 |  | 29 | 33 |
| 2. We use our brains 24 hours a day. | 60 | 74 |  | 30 | 24 |  | 10 | 2 |  | 73 | 73 |  | 14 | 16 |  | 12 | 10 |
| *3. Brain size correlates with intelligence.* | 12 | 12 |  | 84 | 78 |  | 4 | 10 |  | 6 | 8 |  | 88 | 76 |  | 6 | 16 |
| 4. Blind people have better hearing. | 80 | 78 |  | 16 | 20 |  | 4 | 2 |  | 78 | 71 |  | 12 | 16 |  | 10 | 12 |
| *5. Drug use causes holes in your brain.* | 14 | 12 |  | 42 | 50 |  | 44 | 38 |  | 12 | 22 |  | 31 | 37 |  | 57 | 41 |
| *6. Dreaming occurs continuously during sleep.* | **46** | **24** |  | **46** | **66** |  | **8** | **10** |  | 33 | 45 |  | 51 | 49 |  | 16 | 6 |
| 7. The brain is the organ that consumes the most oxygen. | 68 | 62 |  | 6 | 10 |  | 26 | 28 |  | 59 | 67 |  | 16 | 10 |  | 24 | 22 |
| *8. You are born with all of the neurons you will ever have.* | 22 | 22 |  | 50 | 58 |  | 28 | 20 |  | 10 | 12 |  | 59 | 55 |  | 29 | 33 |
| *9. Listening to Mozart’s music makes you smarter.* | 24 | 24 |  | 52 | 48 |  | 24 | 28 |  | 10 | 10 |  | 69 | 63 |  | 20 | 27 |
| *10. Brain and mind are completely independent.* | 26 | 26 |  | 56 | 64 |  | 18 | 10 |  | 10 | 24 |  | 59 | 55 |  | 31 | 20 |
| 11. Memory is stored in networks of cells distributed throughout the brain. | 48 | 58 |  | 8 | 14 |  | 44 | 28 |  | 39 | 45 |  | 4 | 2 |  | 57 | 53 |
| *12. Once you have experienced an event and formed a memory of it, that memory does not change.* | 6 | 4 |  | 88 | 96 |  | 6 | 0 |  | 0 | 4 |  | 98 | 92 |  | 2 | 4 |
| *13. Memory is stored in the brain much like as in a computer. That is, each memory is encoded in a tiny piece of the brain.* | 36 | 54 |  | 38 | 30 |  | 26 | 16 |  | 37 | 53 |  | 18 | 10 |  | 45 | 37 |
| *14. Bilingual education leads to confusion and delayed development, due to conflict between the two language systems.* | 30 | 34 |  | 48 | 56 |  | 22 | 10 |  | 16 | 10 |  | 59 | 65 |  | 24 | 24 |

|  | Experimental Group | | | | | | | |  | Control Group | | | | | | | |
| --- | --- | --- | --- | --- | --- | --- | --- | --- | --- | --- | --- | --- | --- | --- | --- | --- | --- |
|  | Yes (%) | |  | No (%) | |  | I don’t know (%) | |  | Yes (%) | |  | No (%) | |  | I don’t know (%) | |
|  | Pre | Post |  | Pre | Post |  | Pre | Post |  | Pre | Post |  | Pre | Post |  | Pre | Post |
| **II. Brain function (8 statements)** |  |  |  |  |  |  |  |  |  |  |  |  |  |  |  |  |  |
| *1. Brain areas work independently.* | 38 | 28 |  | 50 | 64 |  | 12 | 8 |  | 12 | 8 |  | 69 | 65 |  | 18 | 27 |
| *2. Any brain region can perform any function.* | 12 | 10 |  | 76 | 82 |  | 12 | 8 |  | 10 | 8 |  | 71 | 67 |  | 18 | 24 |
| 3. Language is predominantly processed by the left hemisphere. | 48 | 64 |  | 16 | 10 |  | 36 | 26 |  | 27 | 24 |  | 16 | 14 |  | 57 | 61 |
| *4. Right-hemisphere learners are more creative than left-hemisphere learners.* | 64 | 62 |  | 16 | 18 |  | 20 | 20 |  | **53** | **37** |  | **10** | **29** |  | **35** | **31** |
| 5. The left and right brains are connected by a bundle of nerve fibers called the corpus callosum. | **56** | **80** |  | **0** | **0** |  | **44** | **20** |  | 33 | 47 |  | 2 | 0 |  | 65 | 53 |
| *6. Brief co-ordination exercises can improve integration of left and right hemispheric brain function.* | 70 | 86 |  | 8 | 6 |  | 22 | 8 |  | 57 | 65 |  | 10 | 2 |  | 33 | 33 |
| *7. The left side of the brain deals with rational thinking and the right side is emotional processing.* | 82 | 80 |  | 8 | 10 |  | 10 | 10 |  | 55 | 59 |  | 12 | 6 |  | 33 | 35 |
| 8. The left hemisphere mainly controls the right side of the body and the right hemisphere mainly controls the left side of the body. | 92 | 90 |  | 6 | 6 |  | 2 | 4 |  | 84 | 84 |  | 0 | 2 |  | 16 | 14 |

|  | Experimental Group | | | | | | | |  | Control Group | | | | | | | |
| --- | --- | --- | --- | --- | --- | --- | --- | --- | --- | --- | --- | --- | --- | --- | --- | --- | --- |
|  | Yes (%) | |  | No (%) | |  | I don’t know (%) | |  | Yes (%) | |  | No (%) | |  | I don’t know (%) | |
|  | Pre | Post |  | Pre | Post |  | Pre | Post |  | Pre | Post |  | Pre | Post |  | Pre | Post |
| **III. Brain development (10 statements)** |  |  |  |  |  |  |  |  |  |  |  |  |  |  |  |  |  |
| 1. Brain development involves the birth and  death of neurons. | 60 | 68 |  | 8 | 12 |  | 32 | 20 |  | 67 | 51 |  | 4 | 8 |  | 29 | 41 |
| *2. Brain development has finished by the time  children reach secondary school.* | **10** | **10** |  | **60** | **84** |  | **30** | **6** |  | 4 | 0 |  | 63 | 73 |  | 33 | 24 |
| 3. Brain development occurs earlier for basic  sensorimotor processes such as vision and hearing, and much later for higher cognitive skills such as reasoning and planning*.* | **58** | **82** |  | **4** | **0** |  | **38** | **18** |  | 69 | 57 |  | 2 | 2 |  | 29 | 41 |
| 4. There are sensitive periods in childhood when  it’s easier to learn certain skills. | 80 | 90 |  | 2 | 8 |  | 18 | 2 |  | **71** | **90** |  | **0** | **2** |  | **29** | **8** |
| 5. Teenagers’ impulsive behavior is partially  explained by incomplete brain development. | **36** | **74** |  | **26** | **20** |  | **38** | **6** |  | 43 | 63 |  | 18 | 10 |  | 39 | 27 |
| *6. Environments that provide rich stimuli  improve the brain function of pre-school children.* | 88 | 98 |  | 0 | 2 |  | 12 | 0 |  | 84 | 90 |  | 2 | 2 |  | 14 | 8 |
| 7. The brain continues to develop throughout  adolescence, particularly in the frontal  and parietal cortices. | **42** | **66** |  | **8** | **4** |  | **50** | **30** |  | 45 | 45 |  | 2 | 2 |  | 53 | 53 |
| 8. Myelination, which improves the speed of  neural communication, increases  considerably throughout adolescence. | 44 | 60 |  | 14 | 0 |  | 42 | 40 |  | 37 | 27 |  | 2 | 0 |  | 61 | 73 |
| *9. There are critical periods in childhood after  which certain abilities can no longer develop and certain skills can no longer be learned.* | **66** | **88** |  | **14** | **6** |  | **20** | **6** |  | 55 | 59 |  | 20 | 10 |  | 24 | 31 |
| 10. Circadian rhythms (“body clock”) shift  during adolescence, causing pupils to be tired during the first lessons of the school day. | 48 | 68 |  | 12 | 12 |  | 40 | 20 |  | 41 | 51 |  | 4 | 4 |  | 55 | 45 |

|  | Experimental Group | | | | | | | |  | Control Group | | | | | | | |
| --- | --- | --- | --- | --- | --- | --- | --- | --- | --- | --- | --- | --- | --- | --- | --- | --- | --- |
|  | Yes (%) | |  | No (%) | |  | I don’t know (%) | |  | Yes (%) | |  | No (%) | |  | I don’t know (%) | |
|  | Pre | Post |  | Pre | Post |  | Pre | Post |  | Pre | Post |  | Pre | Post |  | Pre | Post |
| **IV. Brain structure (12 statements)** |  |  |  |  |  |  |  |  |  |  |  |  |  |  |  |  |  |
| *1. Your brain is gray.* | 30 | 38 |  | 30 | 24 |  | 40 | 38 |  | 14 | 24 |  | 22 | 14 |  | 63 | 61 |
| 2. Production of new neural connections can  continue into old age. | 30 | 40 |  | 38 | 30 |  | 32 | 30 |  | 12 | 16 |  | 22 | 24 |  | 65 | 59 |
| *3. The volume of blood in the brain increases  with physical effort.* | 74 | 86 |  | 8 | 4 |  | 18 | 10 |  | 76 | 63 |  | 2 | 4 |  | 22 | 33 |
| *4. A wrinkle in the brain is added each time we learning something new.* | 42 | 42 |  | 24 | 32 |  | 34 | 26 |  | 41 | 29 |  | 22 | 27 |  | 37 | 45 |
| 5. Emotional stimuli are perceived more quickly than neutral stimuli. | 60 | 76 |  | 6 | 4 |  | 34 | 20 |  | 43 | 31 |  | 2 | 8 |  | 53 | 61 |
| *6. Hormones influence the body’s internal state,* ***not*** *a person’s personality.* | 30 | 36 |  | 40 | 42 |  | 30 | 22 |  | 18 | 20 |  | 43 | 45 |  | 39 | 35 |
| 7. Glial cells support neuronal functions and modulate the transmission of signals. | 32 | 40 |  | 2 | 4 |  | 66 | 56 |  | 22 | 22 |  | 0 | 0 |  | 78 | 78 |
| 8. Learning occurs through modification of  the connections between neurons. | 66 | 86 |  | 10 | 2 |  | 24 | 12 |  | 61 | 51 |  | 4 | 6 |  | 35 | 43 |
| 9. When a brain region is damaged, other parts of the brain can sometimes take over its function. | **68** | **62** |  | **14** | **32** |  | **18** | **6** |  | 53 | 57 |  | 20 | 10 |  | 27 | 33 |
| *10. Emotional brain processes interrupt cognitive  brain processes such as thinking and reasoning.* | 26 | 24 |  | 42 | 40 |  | 32 | 36 |  | 18 | 18 |  | 41 | 31 |  | 41 | 49 |
| 11. Each receptor site accepts only certain  neurotransmitters, much like a lock accepts only a certain key. | 56 | 72 |  | 14 | 10 |  | 30 | 18 |  | 37 | 31 |  | 6 | 10 |  | 57 | 59 |
| 12. Communication between different parts of the  brain is through chemical substances and electrical impulses. | 86 | 86 |  | 0 | 6 |  | 14 | 8 |  | 67 | 71 |  | 0 | 2 |  | 33 | 27 |

|  | Experimental Group | | | | | | | |  | Control Group | | | | | | | |
| --- | --- | --- | --- | --- | --- | --- | --- | --- | --- | --- | --- | --- | --- | --- | --- | --- | --- |
|  | Yes (%) | |  | No (%) | |  | I don’t know (%) | |  | Yes (%) | |  | No (%) | |  | I don’t know (%) | |
|  | Pre | Post |  | Pre | Post |  | Pre | Post |  | Pre | Post |  | Pre | Post |  | Pre | Post |
| **V. Neuroimaging (6 statements)** |  |  |  |  |  |  |  |  |  |  |  |  |  |  |  |  |  |
| *1. fMRI can measure the activity of a single neuron.* | 22 | 20 |  | 6 | 14 |  | 72 | 66 |  | 4 | 4 |  | 2 | 8 |  | 94 | 88 |
| 2. Brain activity can be studied through the oxygen  consumption of brain areas. | 58 | 72 |  | 12 | 8 |  | 30 | 20 |  | 55 | 57 |  | 4 | 6 |  | 41 | 37 |
| *3. The failure of an area to “light up” in  neuroimaging means that it has no activity.* | 30 | 28 |  | 38 | 52 |  | 32 | 20 |  | 18 | 27 |  | 39 | 39 |  | 43 | 35 |
| *4. The brain area that is most active is the only  one involved in some cognitive function* | **16** | **22** |  | **38** | **54** |  | **46** | **24** |  | 8 | 2 |  | 53 | 47 |  | 39 | 51 |
| 5. Neuroimaging depicts the correlation between  brain activation and cognitive function. | 52 | 68 |  | 12 | 12 |  | 36 | 20 |  | 41 | 45 |  | 10 | 10 |  | 49 | 45 |
| 6. Brain images are sophisticated reconstructions that depend on complex mathematical assumptions. | **56** | **86** |  | **0** | **4** |  | **44** | **10** |  | 49 | 59 |  | 4 | 4 |  | 47 | 37 |

|  | Experimental Group | | | | | | | |  | Control Group | | | | | | | |
| --- | --- | --- | --- | --- | --- | --- | --- | --- | --- | --- | --- | --- | --- | --- | --- | --- | --- |
|  | Yes (%) | |  | No (%) | |  | I don’t know (%) | |  | Yes (%) | |  | No (%) | |  | I don’t know (%) | |
|  | Pre | Post |  | Pre | Post |  | Pre | Post |  | Pre | Post |  | Pre | Post |  | Pre | Post |
| **VI. Applying neuroscience results (10 statements)** | |  |  |  |  |  |  |  |  |  |  |  |  |  |  |  |  |
| 1. Memory consolidation occurs during dreaming. | 84 | 82 |  | 6 | 2 |  | 10 | 16 |  | 76 | 73 |  | 10 | 6 |  | 14 | 20 |
| *2. “Smart pills” can improve thinking and learning by changing the neurochemistry of the brain.* | 12 | 10 |  | 46 | 52 |  | 42 | 38 |  | 6 | 4 |  | 43 | 39 |  | 51 | 57 |
| 3. Drugs such as cocaine are addictive and affect the mind because they alter the chemical balance of the brain. | 86 | 84 |  | 4 | 8 |  | 10 | 8 |  | 84 | 71 |  | 0 | 0 |  | 16 | 29 |
| *4. Children are less attentive after consuming  sugary drinks and/or snacks.* | 64 | 54 |  | 24 | 20 |  | 12 | 26 |  | **49** | **31** |  | **16** | **24** |  | **35** | **45** |
| *5. If pupils do* ***not*** *drink sufficient amounts of  water (6 to 8 glasses a day) their brains shrink.* | 8 | 4 |  | 50 | 66 |  | 42 | 30 |  | 2 | 6 |  | 49 | 35 |  | 49 | 59 |
| *6. It has been scientifically proven that fatty acid  supplements (omega-3 and omega-6)  have a positive effect on academic achievement.* | 60 | 46 |  | 8 | 12 |  | 32 | 42 |  | 37 | 24 |  | 18 | 22 |  | 45 | 53 |
| 7. ADHD is an illness characterized by inattention  and impulsivity. | 90 | 94 |  | 6 | 0 |  | 4 | 6 |  | 94 | 80 |  | 0 | 2 |  | 6 | 18 |
| *8. Poor parenting is the main cause of ADHD.* | 18 | 16 |  | 50 | 66 |  | 32 | 18 |  | 10 | 14 |  | 63 | 55 |  | 27 | 31 |
| 9. Depression can be caused by the lack of certain  chemicals in the brain. | 62 | 62 |  | 8 | 18 |  | 30 | 20 |  | 41 | 43 |  | 14 | 10 |  | 45 | 45 |
| 10. Diseases such as Parkinson’s and Alzheimer’s are associated with synaptic loss  and cell death in some brain areas. | 58 | 76 |  | 10 | 2 |  | 32 | 22 |  | 65 | 59 |  | 0 | 6 |  | 35 | 35 |

**References.**

Beck DM. The appeal of the brain in the popular press. Perspect Psychol Sci. 2010; 5: 762–6. doi:[10.1177/1745691610388779](http://pps.sagepub.com/content/5/6/762.short)

Dekker S, Lee NC, Howard-Jones P, Jolles J. Neuromyths in education: Prevalence and predictors of misconceptions among teachers. Front Psychol. 2012; 3: 429. doi:[10.3389/fpsyg.2012.00429](http://journal.frontiersin.org/article/10.3389/fpsyg.2012.00429/full)

Genetic Science Learning Center. Learn Genetics [internet] [cited 2013 July 23]. Available from: <http://learn.genetics.utah.edu/content/addiction/>

Herculano-Houzel S. Do you know your brain? A survey on public neuroscience literacy at the closing of the decade of the brain. Neuroscientist. 2002; 8: 98–110. doi:[10.1177/107385840200800206](http://nro.sagepub.com/content/8/2/98.short)

Howard-Jones P. Neuroscience and Education: Issues and opportunities. London: Teaching and Learning Research Programme, ESRC; 2007.

MacNabb C. Schmidt L. Minor S. Roehrig GH. Dubinsky JM. BrainU: The neuroscience teacher institute. [cited 2013 July 23]. Available from <http://brainu.org/brainu-neuroscience-teacher-institute>.

Marcus G. Neuroscience fiction*.* The New Yorker [newspaper on the internet]*.* 2012 Dec 2 [cited 2013 July 23]. Available from: <http://www.newyorker.com/news/news-desk/neuroscience-fiction>

Schultz N. Time to banish the neuromyths in education? New Sci. 2009; 203: 8–9. doi:[10.1016/s0262-4079(09)62446-5](http://www.sciencedirect.com/science/article/pii/S0262407909624465)

Simons DJ, Chabris CF. What people believe about how memory works: A representative survey of the US population. PLoS ONE. 2011; 6**:**e22757. doi:[10.1371/journal.pone.0022757](http://journals.plos.org/plosone/article?id=10.1371/journal.pone.0022757)

Tokuhama-Espinosa T. The new science of teaching and learning: Using the best mind, brain, and education science in the classroom. New York: Teachers College Press; 2010.
